# From Tachycardia to Brain Tumor: An Unusual Evolution of Epilepsy

**DOI:** 10.7759/cureus.92152

**Published:** 2025-09-12

**Authors:** Elwira Misztela-Lisiecka, Grazyna Waska, Anna Strozak, Magdalena A Miernik-Skrzypczak, Piotr Nowakowski

**Affiliations:** 1 Medicine, Pabianice Medical Centre, Pabianice, POL; 2 Internal Medicine, Szpital Specjalistyczny Nr 1 w Bytomiu, Bytom, POL; 3 Internal Medicine, Dr. Anna Gostynska Wolski Hospital, Warsaw, POL; 4 General Medicine, Lower Silesian Center of Oncology, Pulmonology and Hematology, Wrocław, POL; 5 Internal Medicine, Municipal Hospital in Gliwice, Gliwice, POL

**Keywords:** brain tumor, epilepsy, ganglioglioma, hyperprolactinemia, tachycardia

## Abstract

Ganglioglioma is a rare, benign intracranial neoplasm, most commonly observed in the temporal lobes. Prognosis in brain tumor patients depends not only on the degree of malignancy but also on the tumor's location. Patients typically present with refractory epileptic seizures. This report presents a 27-year-old female patient with ganglioglioma, waiting for a correct diagnosis for nearly 10 years. A thorough review of her medical history revealed no identifiable risk factors. A subtotal surgical resection of the tumor was performed, followed by histopathological examination. Currently, four years post-operation, no growth of residual tumor has been observed.

## Introduction

Ganglioglioma is a rare, usually benign, well-circumscribed, often cystic, mixed neuronal-glial tumor (composed of both glial and ganglionic elements), which typically locates in the temporal lobe and rarely invades surrounding tissues [1-2]. The described patient initially presented with supraventricular tachycardia and was initially diagnosed and treated for cardiological issues, with an incorrect diagnosis of vasovagal syncope of the vasodepressor type. The patient also had hyperprolactinemia that was resistant to treatment. Subsequently, she was referred to a psychiatrist due to symptoms of derealization and depersonalization. Antidepressant therapy led to the diagnosis of a brain tumor. This case illustrates an atypical diagnostic pathway leading to the recognition of a rare CNS tumor and highlights the importance of a comprehensive differential diagnosis. This is why this case is worth describing.

## Case presentation

The patient experienced tachycardia occurring two to three times a week. She had no other symptoms such as dizziness, weakness, shortness of breath, or syncope during or before the attack. Each attack lasted approximately 60 seconds and resolved spontaneously. She had no chronic illnesses. Laboratory tests were ordered with a focus on thyroid hormones. Propranolol was prescribed to manage tachycardia. Results were within normal range. Symptoms persisted. The patient was referred to a cardiologist. During the specialist visit, an ECG was performed, with a normal result. The consultation did not reveal any abnormalities, and increased physical activity was recommended. The symptoms continued, so the patient returned to another cardiologist after several weeks. The ECG was repeated, and the diagnostic workup was expanded to include an echocardiogram. Tests again showed no abnormalities. Reduced physical activity was recommended. After three years, during a routine visit to the family doctor, the patient experienced a tachycardia attack in the office. During the attack, the heart rate was regular at 160/min, blood pressure was 110/75 mmHg, and the lung sounds were normal. An ECG was quickly ordered, but the attack had resolved by the time the patient reached the examination room. She was referred to the electrocardiology ward for ablation, with suspicion of atrioventricular nodal reentrant tachycardia (AVNRT). During hospitalization, an electrophysiological study was performed. The study ruled out the presence of an accessory conduction pathway. A tilt table test was performed. The entire test lasted 35 minutes, with the table inclined at 70 degrees, starting with a 10-minute supine position. Results are shown in Table 1.

**Table 1:** Results of the tilt table test showing sudden hypotension and mild bradycardia at 15 minutes.

Time (min)	Heart rate (beats/min)	Blood pressure (mmHg)	Symptoms
0 (resting)	72	125/78	none
5	74	123/76	none
10	76	122/74	none
15	62	72/38	dizziness, pallor, cold sweat, syncope

After 15 minutes in the upright position, the patient reported sudden weakness, dizziness, nausea, cold sweats, and blurred vision. A rapid drop in blood pressure (to 72/38 mmHg) was observed, with no significant bradycardia (heart rate 62/min). Upon lowering the table to a supine position, vital signs gradually returned to normal within two minutes. The patient was discharged with a diagnosis of vasovagal syncope of the vasodepressor type, with a sudden drop in blood pressure and a minor decrease in heart rate as the predominant component. Physical activity and increased fluid intake were recommended.

In the meantime, the patient underwent regular gynecological checkups. In subsequent laboratory tests, prolactin levels ranged from 82.6 ng/ml to 108.4 ng/ml. She underwent multiple courses of cabergoline and bromocriptine therapy. During treatment, prolactin levels returned to the reference range, only to rise again after discontinuing the medications. A computed tomography scan of the head did not reveal an enlarged pituitary gland.

Nine years after the first symptoms appeared, in addition to persistent paroxysmal tachycardia, the patient began experiencing new, disturbing symptoms. She described two phenomena: one as a feeling of unreality and being in a movie, and the other as a sensation of being detached from her body. The patient was referred for psychiatric evaluation. In the meantime, she noticed anisocoria. The psychiatrist diagnosed anxiety disorders and initiated escitalopram therapy.

One month after starting selective serotonin reuptake inhibitor (SSRI) treatment, the patient experienced a seizure in the form of a tonic-clonic absence seizure. The seizure was reported to the patient by witnesses, as she did not remember the event. According to their description, the patient had her gaze fixed on one spot, was chewing her lips, and moved her hands in an inconsistent and unnatural manner.

**Figure 1:**
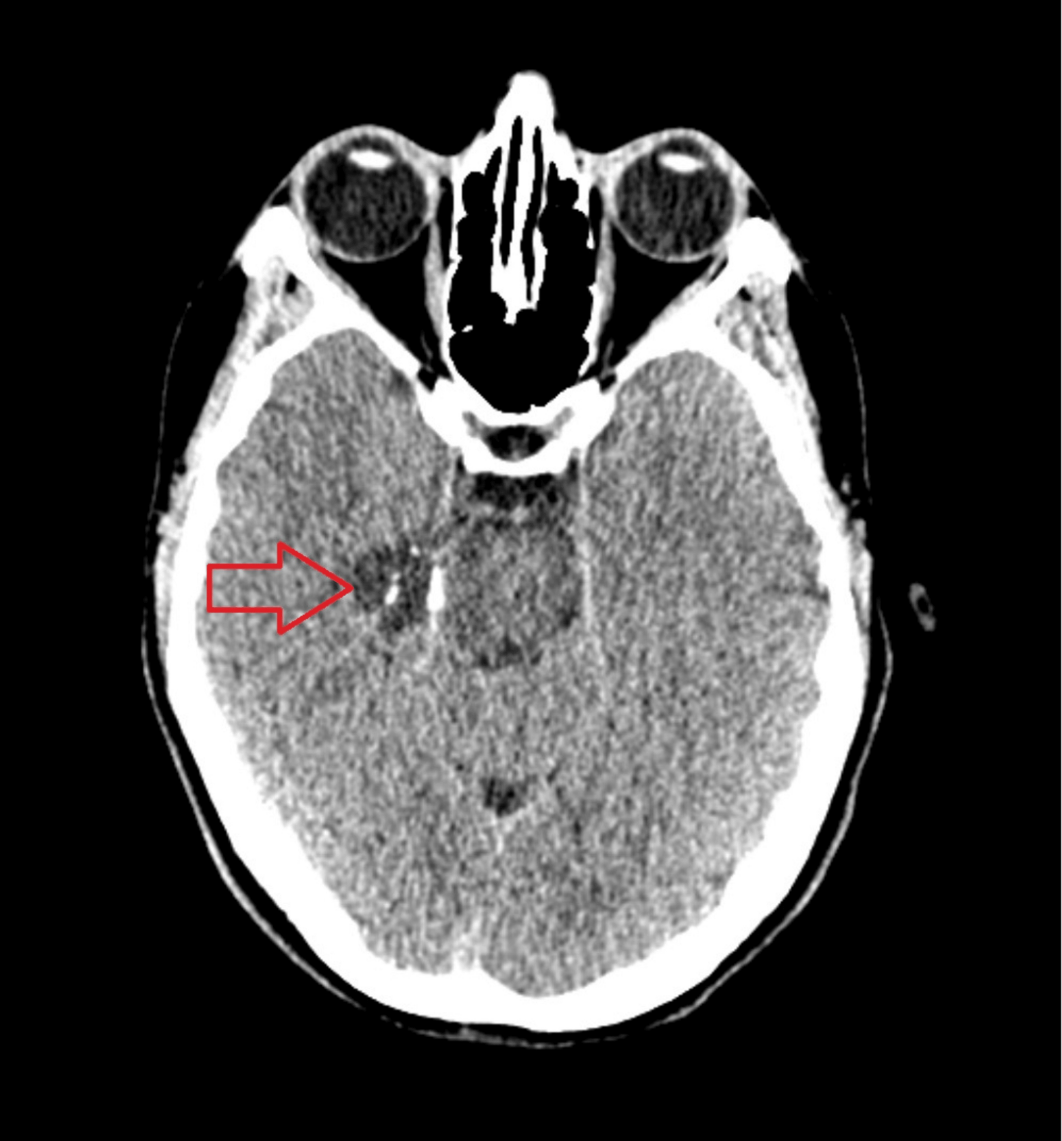
CT showing a newly identified suspicious cyst not previously described

Physicians recommended re-examining the computed tomography (CT) scan of the head performed during the diagnosis of hyperprolactinemia. A neurologist and radiologist identified a suspicious cyst that had not been previously described (Figure 1). 

**Figure 2:**
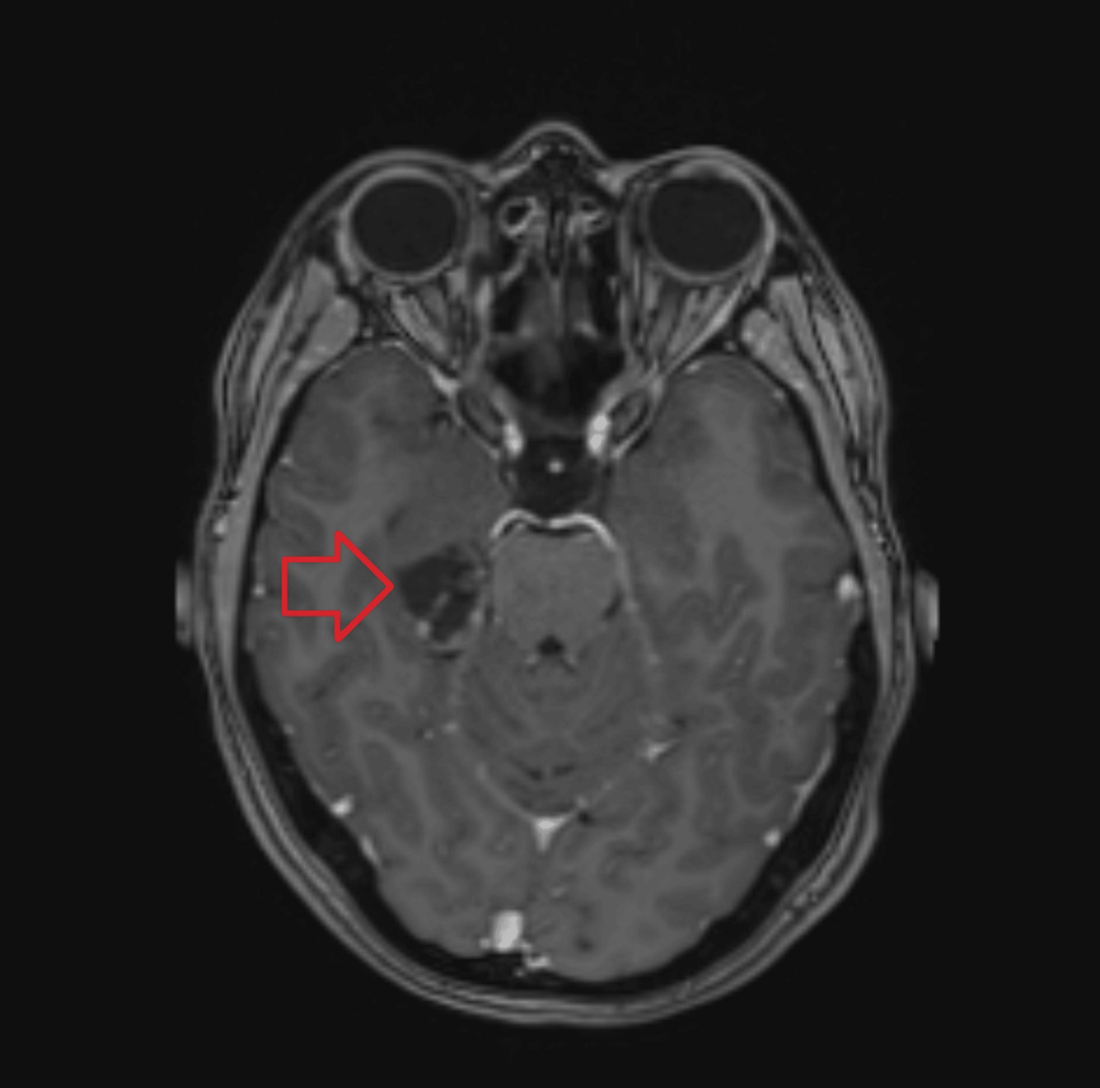
MRI of the right temporal lobe showing a lesion posterior to the hippocampus suggestive of pilocytic astrocytoma

During hospitalization in the neurology ward, the diagnostic workup was expanded. An EEG was performed, revealing temporal lobe epilepsy, and an MRI of the head was conducted, leading to suspicion of a pilocytic astrocytoma located in the right temporal lobe posterior to the hippocampus (Figure 2).

Antiepileptic drugs were initiated, and the patient was referred for neurosurgical treatment. The tumor was not completely removed due to its location, as it was adjacent to the brainstem and resting on the carotid artery. Histopathological examination with immunohistochemical staining ruled out a pilocytic astrocytoma, and the final diagnosis was ganglioglioma. Antiepileptic therapy resolved the paroxysmal tachycardia, and prolactin levels have remained within the established normal range. Three years after surgery, a follow-up MRI of the head showed no evolution of the residual tumor fragments.

## Discussion

The presented case illustrates diagnostic difficulties arising from nonspecific symptoms that initially suggested cardiological causes of arrhythmias, ultimately proven to be manifestations of a central nervous system tumor. Persistent paroxysmal episodes of tachycardia over many years, in the absence of clear abnormalities in cardiovascular imaging and electrophysiological studies, indicated the need to expand the diagnostic workup to include non-cardiological, including neurological and psychiatric, etiologies. In the described case, tachycardia attacks were initially classified as a potential AVNRT, but an electrophysiological study did not confirm the presence of pathological conduction pathways. Further diagnostic workup included a tilt table test, which demonstrated a typical pattern of vasovagal syncope of the vasodepressor type - common in young women and often incorrectly considered the sole cause of symptoms [3]. However, it is essential to emphasize that the presence of vasovagal syncope does not exclude the coexistence of other pathologies, particularly in cases of nonspecific and treatment-resistant symptoms.

It is worth noting the long-standing course of hyperprolactinemia, demonstrating dependence on dopamine agonist treatment and recurrence upon discontinuation. The absence of focal changes in the pituitary gland on computed tomography suggested idiopathic hyperprolactinemia, but it is essential to highlight the limited sensitivity of CT in evaluating intracranial structures, particularly the hypothalamic-pituitary area and temporal lobes [4,5].

The turning point in the diagnostic course was a seizure episode manifesting as psychomotor automatisms and absence seizures, characteristic of temporal lobe epilepsy. SSRIs, including the escitalopram taken by the patient, lower the seizure threshold and increase the risk of epileptic seizure occurrence [6]. The typical description of the patient's behavior by witnesses, her lack of awareness of the event, and previous symptoms of depersonalization and derealization suggest a focal origin of seizures from the temporal lobe. EEG confirmed the diagnosis, and an MRI of the head revealed a lesion of tumor type, ultimately diagnosed as ganglioglioma - a rare, slow-growing benign tumor, often located in the temporal lobe and manifesting as drug-resistant epilepsy.

The relationship between the presence of the tumor and cardiological symptoms can be explained by the functional interaction of the epileptogenic focus with the autonomic nervous system, particularly through limbic structures (hippocampus, amygdala) and their connections with the brainstem and solitary nucleus. These mechanisms may lead to both tachyarrhythmias and vasodepression or bradycardia, depending on the nature of bioelectrical activity and the focus location [7]. The literature describes numerous cases of arrhythmias, including sudden cardiac death, as manifestations of temporal lobe epilepsy (sudden unexpected death in epilepsy (SUDEP)) [8,9].

The resolution of paroxysmal tachycardia after initiating effective antiepileptic therapy and surgical removal of a portion of the tumor provides strong evidence for the cerebral cortex as the primary generator of autonomic symptoms in this case. Stabilization of prolactin levels after antiepileptic treatment further suggests a possible influence of epileptogenic activity on hormonal regulation through the limbic system and hypothalamus.

The conclusions from this case should encourage a broad and multidisciplinary diagnostic approach in situations of atypical autonomic symptoms, particularly in young patients, in whom the cause cannot be established despite advanced cardiological and endocrinological evaluation. Symptoms such as paroxysmal tachycardia, derealization, hormonal fluctuations, and subsequent seizures may share a common denominator in the form of an epileptogenic focus or a tumor in brain structures responsible for autonomic and neuroendocrine regulation.

In conclusion, this case highlights the importance of considering neurogenic causes in persistent autonomic symptoms, especially when standard cardiological and endocrinological workups are inconclusive.

## Conclusions

This case highlights the necessity of an interdisciplinary diagnostic approach in patients with atypical, multisystemic symptoms. Paroxysmal tachycardia, initially misdiagnosed as a functional arrhythmia, was revealed to be temporal lobe epilepsy secondary to a ganglioglioma. Chronic hyperprolactinemia and dissociative symptoms indicated possible temporal and diencephalic involvement. Symptom resolution following antiepileptic treatment and partial tumor resection confirms causality. Persistent autonomic symptoms with a normal cardiological and endocrinological workup should prompt consideration of neurological causes, including focal epilepsy and brain tumors, especially when psychiatric and hormonal abnormalities coexist.
